# Purification, Characterization and *in vitro* Anti-Tumor Activity of Proteins from *Arca subcrenata* Lischke

**DOI:** 10.3390/md20080020

**Published:** 2008-07-10

**Authors:** Liyan Song, Shengfang Ren, Rongmin Yu, Chunyan Yan, Tingfei Li, Yu Zhao

**Affiliations:** 1 College of Pharmaceutical Sciences, Zhejiang University, Hangzhou 310058, P.R. China; 2 College of Pharmacy, Jinan University, Guangzhou 510632, P.R. China

**Keywords:** *Arca subcrenata* Lischke, protein, purification, *in vitro* anti-tumor activity

## Abstract

Two purified proteins G-6 and G-4-2 were obtained from *Arca subcrenata* Lischke using the homogenization, salting-out with ammonium sulfate, ion-exchange chromatography and gel filtration chromatography techniques. The purity of G-6 and G-4-2 was over 96%, as measured by RP-HPLC. G-6 and G-4-2 were measured by SDS-PAGE and IEF-PAGE to have molecular weights of 8.2 kDa and 16.0 kDa, and isoelectric points of 6.6 and 6.1, respectively. The amino acid constituents of G-6 and G-4-2 were also determined. The existence of saccharides in G-6 was demonstrated by the phenol-sulfuric acid method. G-6 and G-4-2 inhibited the proliferation of human tumor cells *in vitro*. By MTT assay, the IC_50_ values of G-4-2 were 22.9 μg/mL, 46.1 μg/mL and 57.7 μg/mL against Hela, HL-60 and KB cell lines, respectively, and the IC_50_ value of G-6 against HL-60 cell line was measured to be 123.2 μg/mL.

## 1. Introduction

In recent years, more and more researchers have come to the realization that marine organisms hold immense potential as a source of novel molecules and new anticancer agents. Because of their longer evolutionary history, marine organisms likely possess a greater molecular diversity than do their terrestrial counterparts [1–2]. Over the past two decades, a global collaborative effort has been raised that aims at the discovery of novel and clinically useful anti-tumor agents derived from marine organisms [3–7]. According to existing literature, more than ten new experimental anti-tumor agents derived from marine sources have entered clinical trials [8], including bryostatin-1, aplidine, ecteinascidin-743 (ET-743) [2], Kahalalide F [9], as well as derivatives of dolastatin such as TZT-1027 [10] and LU 103793 [11].

*Arca subcrenata Lischke* is a marine invertebrate that belongs to Arcidae family under Phylum Mollusca, Class Lamellibranchiata. A Chinese Traditional Material, wa leng zi (*Concha Arcae*), is made from *A. subcrenata*. As a marine mollusk native to the seas around China, *A. subcrenata* has been utilized in the treatments of tumor, anemia and inflammation for centuries in Chinese Traditional medicine [12]. The concentration of protein and saccharide, proportions of amino acid, ultraviolet spectrum analysis and trace element analysis of *A. subcrenata* have been previously reported [13]. Its hydrolysate was found to possess hypoglycemic activity on mice with alloxan-induced hyperglycemia, hypolipidic activity in experimental mouse model of hyperlipidemia [14], as well as protective effects on liver injuries in mice [15]. More recently, polysaccharide from *A. subcrenata* was demonstrated to induce proliferation of spleen lymphocyte *in vitro* [16]. However, no information is currently available on its anti-tumor activity either *in vitro* or *in vivo*. During the course of our extensive screening program on marine traditional Chinese medicines for *in vitro* anti-tumor activities, *A. subcrenata* was selected because of its widespread geographic distribution in China and its unique reputation in folklore medicine.

In our present study, purification and characterization of protein components of *A. subcrenata* were investigated. Their *in vitro* anti-tumor activity was also evaluated by examining cytotoxicity against seven human tumor cell lines.

## 2. Materials and Methods

### 2.1 Materials

DEAE Sepharose Fast Flow and Sephadex G-50 were obtained from Pharmacia Biotechnology. L-glutamide and 3-(4,5-dimethylthiazol-2-yl)-2,5-diphenyltetrazolium bromide (MTT) were purchased from Amresco Inc., Solon, Ohio, USA. Coomassie Brilliant Blue G-250 and Bovine Serum Albumin were obtained from Sino-American Biotechnology Co., USA. RPMI-1640 medium was purchased from GIBCO, USA and Cisplatin (cis-diamminedichloroplatinum, CDDP) from David Bull Laboratory. Fetal bovine serum (FBS) was provided by Hangzhou Sijiqing Corp, P.R. China. Benzylpenicillin sodium was obtained from Jiangxi Dongfeng Pharmaceutical Co., Ltd., P.R. China. Streptomycin sulfate was produced by North China Pharmaceutical Group Corporation. Molecular weight markers were obtained from Shanghai Puyi Biotechnology Co., Ltd., P.R. China. All other chemicals and reagents used were of analytical grade.

### 2.2 Extraction of total proteins

The visceral mass of *A. subcrenata* (200 g) was washed with 4 °C tap water three times, followed by washing with 4 °C distilled water three times. It was then homogenized with double volume of PBS (10 mM, pH 8.0) at 4 °C for 3 min. After centrifugation (16,000 g, 20 min) at 4 °C, the supernatant was collected and the crude extract obtained. The crude extract of *A. subcrenata* was fractionated by salting out with increasing concentrations of ammonium sulfate. Solid ammonium sulfate was slowly added to the above crude extract with gentle stirring, up to 35% saturation in 20 min. After the crude extract was left at 4 °C with the ammonium sulfate under vortexing for another 40 min [17], the protein precipitate was collected by centrifugation (16,000 g, 20 min) at 4°C. The supernatant was transferred to another beaker, and solid ammonium sulfate was added to it up to 70% saturation. The mixture was treated as above. Likewise, another protein precipitate was obtained at 70% 100% saturation of ammonium sulfate. Each of the three protein pellets was suspended in 10 ml of ice-cold PBS (10 mM, pH 8.0), and dialyzed against a large volume (3 L) of distilled water for 24 h at 4 °C. Dialysis bags, with molecular weight cut-off (MWCO) of 1000 Da (Spectro/Pro 6: Spectrum-Laboratories, Inc.), were employed. During this process, the dialysate was changed three times to completely remove any residual ammonium sulfate.

### 2.3 Purification of proteins

#### *Anion exchange chromatography* (DEAE Sepharose Fast Flow)

Fraction III obtained from the crude extract at 70%–100% saturation of ammonium sulfate was dialyzed against 10 mM Tris-HCl, pH 7.46 for 5 h and the dialyzed solution was subsequently injected into a DEAE Sepharose Fast Flow column, which was pre-equilibrated with the aforementioned Tris-HCl buffer. The column was washed with the same buffer until the baseline returned to zero and remained stable. The column was then eluted with increasing concentration of NaCl prepared in 10 mM Tris-HCl buffer, pH 7.46 at 4 °C. Aliquots of 5 ml/tube were collected at a flow rate of 1.2 ml/min and the absorbance was measured at 280 nm. Seven A_280 nm_ peak fractions, named G-1, G-2, G-3, G-4, G-5, G-6 and G-7, were collected respectively.

#### *Gel filtration chromatography* (Sephadex G-50)

The protein solution that passed through the anion exchange chromatographic column was concentrated by freeze-drying and loaded onto a Sephadex G-50 column that was pre-equilibrated with 10 mM Tris-HCl buffer, pH 7.46. The flow rate was 0.65 ml/min, and the absorbance was monitored at 280 nm. Three A_280 nm_ peak fractions, termed as G-4-1, G-4-2 and G-4-3, were obtained respectively.

### 2.4 Cytotoxicity assay

#### Cell lines and culture

Tumor cell lines used in this study included seven human cell lines, namely A549 (lung cancer cells), BEL-7404 (hepatocellular carcinoma cells), CNE (nasopharyngeal carcinoma cells), Hela (cervical carcinoma cells), PC-3 (prostatic carcinoma cells), HL-60 (leukemia cells) and KB (oral epithelial cancer cells). All of cell lines were provided by Shanghai Institutes for Biological Sciences, Chinese Academy of Sciences, and the cells were cultured in RPMI 1640 medium supplemented with heat-inactivated 10% FBS, 0.2 mg/L L-glutamide, 1.0 mg/mL NaHCO_3_, 100 units/mL penicillin, and 100 units/mL streptomycin in a humidified incubator at 37 °C and 5% CO_2_ atmosphere.

#### Evaluation of cytotoxicity

Cultured cells were taken during their exponential growth phase. Routinely, the cells were detached with 0.25% trypsin and cell suspension was made in the above medium. By trypan blue exclusion, cell viability greater than 95% was determined. One hundred microliters of cell suspensions containing 5 × 10^3^ cells were seeded in each well of a 96-well microtiter plate and incubated at 37 °C in a humidified incubator with 5% CO_2_. After 24 h, the cells were treated with aseptic samples which were sterilized by passing through 0.22 μm Millipore filter, and CDDP was used as the positive control. Untreated cells were used as negative control. Each plate was incubated for another 72 h at 37 °C in a humidified incubator with 5% CO_2_. Cytotoxic activity was evaluated *in vitro* by MTT assay [18] and is expressed as IC_50_, i.e. the sample concentration able to inhibit cell growth by 50% compared with the untreated control. All experiments were carried out in triplicate, and data in the form of mean ± SD are presented.

### 2.5 Protein assay

The protein content of total protein extract, fraction-I, fraction-II, fraction-III, G-6, G-4 and G-4-2 was determined by the method of Bradford [19] with bovine serum albumin as standard.

### 2.6 Sodium dodecyl sulfate-polyacrylamide gel electrophoresis (SDS-PAGE)

The Sodium dodecyl sulfate*-*polyacrylamide gel electrophoriesis (SDS-PAGE) analysis was performed with a mini-gel apparatus (Protean II. BioRAD, USA). There were two SDS- PAGE systems employed. The samples were analyzed by SDS-PAGE in Laemmli gel [20] with an acrylamide concentration of 5% for the stacking gel and 12% for the running gel, as well as in Tricine-SDS-PAGE [21] with an acrylamide concentration of 4% for the stacking gel, 10% for the space gel and 15.5% for the running gel. Five to thirty micrograms of protein per well was loaded on the gel. Protein bands were detected by the Coomassie blue staining method [22].

### 2.7 Isoelectric focusing-polyacrylamide gel electrophoresis (IEF)

Ampholyte (40%, pH 3.5–10.0) was used to prepare IEF gel with acrylamide concentration of 5%. The isoelectric focusing-polyacrylamide gel electrophoresis (IEF-PAGE) was carried out on BIO-RAD power PAC-300 and Mini-PROTEAN 3 cell provided by BIO-RADKWS at 150 V for 75 volt-hours (vh), then at 200 V for 500 vh. The IEF-PAGE gel unloaded samples was washed by double distilled water, and sliced into pieces of 0.5 cm in length from acidic terminal to basic terminal, then separately dipped into Eppendorf tubes containing 2.0 mL of 10 mM KCl for 30 min. The pH value of the liquid around each slice was measured. The gel-loaded samples was fixed with 10% trichloroacetic acid for 30 min, and rinsed thoroughly with destaining solution (0.25% SDS, 33% ethanol, and 10% acetic acid). Data were derived from calibration curve of isoelectric point with the length of gel as abscissa and pH value as ordinate.

### 2.8 Reversed-phase high performance liquid chromatography (HPLC)

HPLC analyses were performed on an Agilent series 1100 HPLC system fitted with a reversed-phase high performance liquid chromatography (RP-HPLC) cartridge, 4.6 mm × 150 mm filled with ZORBAX® 300SB-C8, 5 μm (Agilent). The solvent system was the following: solvent A: 0.1% (v/v) trifluoroacetic acid (TFA) in H_2_O; solvent B: 0.1% (v/v) TFA in H_2_O: acetonitrile 1: 4 (v/v) [23–28]. Gradient elution from 50% to 70% of solvent B in 35 min; flow rate: 1 mL/min; detection wavelength: 280 nm; column temperature 30 °C.

### 2.9 Analysis of saccharides

The concentrations of methyl glycoproteins and glycoproteins were accurately determined by using calibration curves composed of the appropriate monosaccharide(s) obtained with a modified version of the colorimetric phenol-sulfuric acid method [29–31]. 200 μg/mL glucose solution was served as standard. The absorbance at 490 nm was used to determine the amount of carbohydrate in the sample.

### 2.10 Analysis of amino acid components

The content of amino acids was analyzed by an amino acid automatic analyzer (Hitachi 835–50) fitted with ion exchange resin (Hitachi 2619). Appropriate amount of purified and freeze-dried samples were weighed and applied into hydrolysis tube. 6 mol/L HCl was added and the vessel was sealed and evacuated. Hydrolysis was allowed to proceed at 110°C for 24 h. The samples were then dried, dissolved in 0.02 N HCl, and centrifuged at 10,000 rpm for 15 mins. The amino acid compositions were then obtained by automatic analysis algorithm of Hitachi L 835–50. The analysis conditions were as follows: flow rate: 0.225 mL/min, column temperature: 53°C, column pressure: 80–130 kg/cm^2^, analysis time: 74 min.

## 3. Results and Discussion

### 3.1 In vitro bioactivity-guided fractionation

Cytotoxicity is one of the chemotherapeutic hallmarks of anti-tumor activity [32]. MTT assay, a well-established *in vitro* model for cytotoxicity against cancer cell lines, was used as one of conventional methods for the screening of compounds with potential anti-tumor properties [33]. Since it is well known that different cell lines can exhibit different sensitivities to a cytotoxic compound, seven tumor cell lines with different origins, morphologies, and tumorigenicities were employed.

To screen natural products with *in vitro* anti-tumor activity, the total proteins extract of *A. subcrenata* was subjected to cytotoxicity assay. The results showed that the total proteins extract of *A. subcrenata* suppressed the proliferation of Hela, HL-60, CNE and A549 cells, with IC_50_ values of 35.6 μg/mL, 106.0 μg/mL, 145.9 μg/mL and 306.1 μg/mL, respectively (Table 1).

In order to identify cytotoxic components, the total proteins extract of *A. subcrenata* was fractionated by salting-out at increasing saturation levels of ammonium sulfate. The Fraction-I, Fraction-II, and Fraction-III were obtained at the ammonium sulfate saturation of 0–35%, 35–70% and 70–100%, and their percentage yield was 7.1%, 41.4% and 25.4%, respectively. In the evaluation of cytotoxicity of three fractions, the fraction-III exhibited the significant inhibition on the proliferation of Hela, HL-60 and KB cells with IC_50_ values of 6.7 μg/mL, 14.7 μg/mL and 76.5 μg/mL, respectively (Table 1). The results suggested that the fraction-III warranted further purification in order to unveil the active components of *A. subcrenata*.

### 3.2 Purification of cytotoxic proteins from A. subcrenata

**Figure 1:**
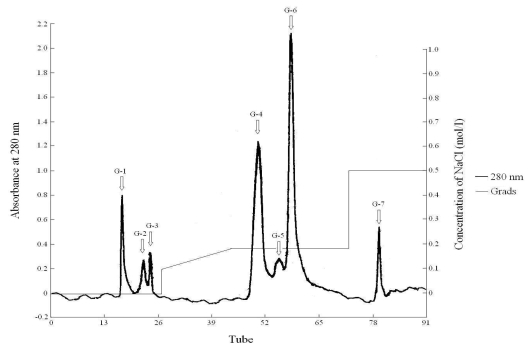
The curve of elution in DEAE Sepharose Fast Flow chromatography Column specification: 1.6 × 30 cm; Equilibrate liquid: buffer C (Tris-HCl, pH 7.46, 10 mM); Sample: Fraction-III; Detection wavelength: UV 280 nm; Flow rate: 1.2 mL/min; Collection rate: 5 mL/tube.

Fraction-III was subjected to ion exchange chromatography for purification. Seven A_280 nm_ peak fractions were obtained through elution with increasing concentrations of NaCl containing Tris-HCl (10 mM, pH 7.46) on DEAE Sepharose Fast Flow column (Figure 1).

The results of cytotoxicity experiments showed that fraction G-6 had the ability to only suppress the proliferation of HL-60 cells (IC_50_ = 123.2 μg/mL), whereas fraction G-4 appeared to possess inhibitory activity not only against HL-60 cells (IC_50_ = 67.8 μg/mL), but also against Hela cells (IC_50_ = 38.2 μg/ml) and KB cells (IC_50_ = 78.1 μg/mL).

**Figure 2:**
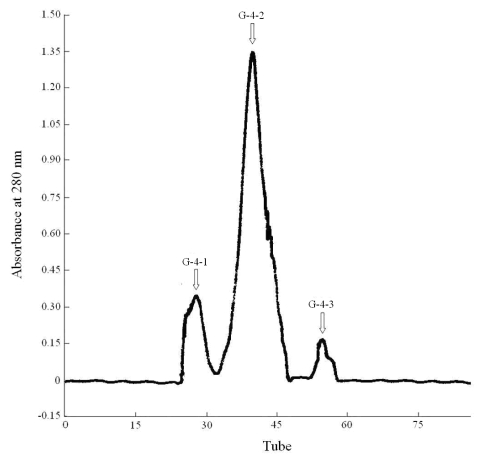
The curve of G-4 separated by Sephadex G-50 chromatography Column specification: 1.0 × 100 cm; Equilibrate liquid: buffer C (Tris-HCl, pH 7.46, 10 mM); Sample: G-4; Detection wavelength: UV 280 nm; Flow rate: 0.65 mL/min; Collection rate: 3 mL/tube.

Furthermore, fraction G-4 was rechromatographed on a Sephadex G-50 column (Figure 2). The other subfractions were not found to be active against these seven human tumor cell lines.

Three A_280 nm_ peak fractions were collected and evaluated for their cytotoxic activities. Among the fractions, the IC_50_ values of fraction G-4-2 towards Hela, HL-60 and KB cell lines were measured to be 22.9 μg/mL, 46.1 μg/ml, and 57.7 μg/mL, respectively.

Fraction-III showed cytotoxic activity against A549, but none of its subfractions presented that activity. There are probably synergistic and additive effects among subfractions in the fractionation of natural marine medicines. These effects weaken as further isolation and purification proceeds. This is corroborated by the observation that each subfraction of fraction-III exhibits lower activity towards A549 than fraction-III. In the same way fraction-III presented a lower IC_50_ than some of its subfractions (i.e. against Hela and HL-60 lines), and purified subfractions showed lower activities against Hela and HL-60 lines.

### 3.3 Characterization of purified proteins

**Figure 3:**
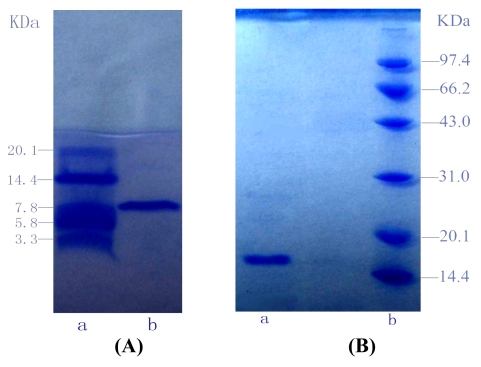
The SDS-PAGE of G-6 and G-4-2 (A) Tricine-glycerol SDS-PAGE of G-6. lane a: molecular weight marker (range from 3.3 kDa to 20.1 kDa); lane b: G-6. (B) SDS-PAGE of G-4-2. lane a: G-4-2; lane b: molecular weight marker (range from 14.4 kDa to 97.4 kDa)

To estimate the molecular weight of G-6, Tricine-glycerol SDS-PAGE was employed with molecular weight marker ranging from 3.3 kDa to 20.1 kDa as standard. Similarly, SDS-PAGE was utilized to estimate the molecular weight of G-4-2, with molecular weight marker ranging from 14.4 kDa to 97.4 kDa as standard (Figure 3).

**Figure 4:**
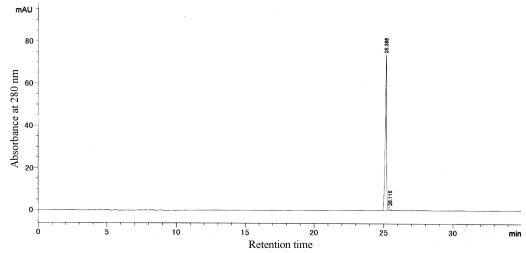
RP-HPLC of G-6.

**Figure 5:**
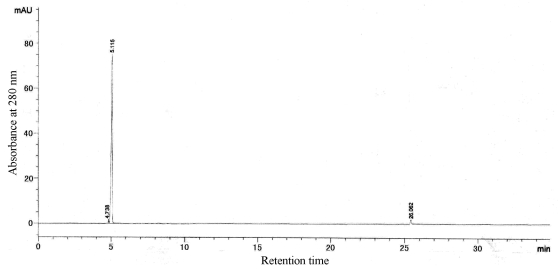
RP-HPLC of G-4-2.

According to calibration curves, the molecular weights of G-6 and G-4-2 were 8.2 kDa and 16.0 kDa. And the isoelectric points (pI) of G-6 and G-4-2 were 6.6 and 6.1, respectively. The two proteins were both single bands in SDS-PAGE and IEF-PAGE. Furthermore, the results of RP-HPLC (Figure 4 and Figure 5) indicated the purity of G-6 and G-4-2 was 97.6% and 96.8%, respectively.

According to modified version of the colorimetric phenol-sulfuric acid method, G-6 may be a glycoprotein but not G-4-2. The amino acid compositions of G-6 and G-4-2 were shown in Table 2 and Table 3.

## 4. Conclusions

Based upon the results of the *in vitro* cytotoxicity assay, two proteins from *A. subcrenata* underwent the bioactivity-guided fractionation and purification. Important characteristics of proteins G-6 and G-4-2 were identified, showing their molecular weights to be 8.2 kDa and 16.0 kDa and their isoelectric points to be 6.6 and 6.1, respectively. G-6, but not G-4-2, is shown to be a glycopeptide.

Additionally, our present study reveals for the first time the *in vitro* anti-tumor activity of *A. subcrenata*, and the significance of this finding is deepened by the knowledge that in the past, many pharmaceutical agents were discovered by screening natural products from plants, animals, marine organisms and microorganisms [34]. *A. subcrenata* is a member of mollusks constituting a large marine animal family. Some mollusks such as oyster have been found to contain compounds with anti-tumor activities. The extract of oyster has significant *in vitro* cytotoxic effect on human A549 cell lines [35], and *in vivo* anti-tumor activity against murine hepatic cancer [36]. In addition, there has been much interest in the research on bioactive peptides from marine sources. For example, protein from Chlamys farreri (PCF), a species of Chinese scallop, was recently shown to have potential antioxidant activity and protective effect against ultraviolet (UV) irradiation [37]. Similarly, a group in Korea reported that methanolic extracts from seaweed Plocamium telfairiae (PTE) exhibited a cytotoxic effect against HT-29 human colon carcinoma cells [38].

The combination of chemical and biological approaches provides scientists with a valuable tool to efficiently investigate a wide array of natural products. As a whole, the results in our paper suggest that *A. subcrenata* is a promising resource in the development of novel drugs for its anti-tumor potential. Further studies on mechanism of bioactive proteins, their structure-function relationships and the *in vivo* anti-tumor effects of *A. subcrenata* are currently underway.

## Figures and Tables

**Table 1:** Cytotoxicity of protein samples against seven human tumor cell lines (IC_50_ μg/mL ± SD, n = 3).

Samples	Cell lines						
	A549	Hela	PC-3	HL-60	KB	BEL-7404	CNE
Total protein extract	306.1±28.4	35.6±2.9	>500	106.0±14.6	>500	>500	145.9±25.9
Fraction-I	>500	>500	>500	>500	>500	>500	>500
Fraction-II	>500	>500	>500	>500	>500	>500	>500
Fraction-III	351.7±40.6	6.7±0.8	>500	14.7±1.7	76.5±4.9	>500	>500
G-6	>500	>500	>500	123.2±11.3	>500	>500	>500
G-4	>500	38.2±2.7	>500	67.8±7.1	78.1±9.0	>500	>500
G-4-2	>500	22.9±2.4	>500	46.1±3.5	57.7±7.2	>500	>500

Fraction-I: (0–35% saturated (NH_4_)_2_SO_4_); Fraction-II: (35–70% saturated (NH_4_)_2_SO_4_); Fraction-III: (70–100% saturated (NH_4_)_2_SO_4_)

**Table 2:** The amino acid compositions of G-6.

Amino acid	The content of amino acid(mg/ml)	The mass percentage of amino acid (%)
Asp	0.0352	11.72
Thr	0.0160	5.33
Ser	0.0000	0.00
Glu	0.0426	14.18
Gly	0.0102	3.39
Ala	0.0178	5.93
Cys	0.0483	16.08
Val	0.0123	4.09
Met	0.0056	1.86
Ile	0.0079	2.63
Leu	0.0142	4.73
Tyr	0.0157	5.23
Phe	0.0039	1.30
Lys	0.0251	8.36
His	0.0214	7.12
Arg	0.0197	6.56
Pro	0.0000	0.00

**Table 3:** The amino acid compositions of G-4-2.

Amino acid	The content of amino acid(mg/ml)	The mass percentage of amino acid (%)
Asp	0.2563	15.64
Thr	0.1005	6.13
Ser	0.0633	3.86
Glu	0.2364	14.43
Gly	0.0741	4.52
Ala	0.0747	4.56
Cys	0.0440	2.69
Val	0.0657	4.01
Met	0.0613	3.74
Ile	0.1035	6.32
Leu	0.0412	2.51
Tyr	0.0758	4.63
Phe	0.1056	6.45
Lys	0.2100	12.82
His	0.0335	2.04
Arg	0.0426	2.60
Pro	0.0000	0.00
